# Accurate and robust prediction of Amyloid-β brain deposition from plasma biomarkers and clinical information using machine learning

**DOI:** 10.3389/fnagi.2025.1559459

**Published:** 2025-08-18

**Authors:** Jiayuan Xu, Andrew J. Doig, Sofia Michopoulou, Petroula Proitsi, Fumie Costen, The Alzheimer's disease neuroimaging initiative

**Affiliations:** ^1^Department of Electrical and Electronic Engineering, University of Manchester, Manchester, United Kingdom; ^2^Division of Neuroscience, Stopford Building, School of Biological Sciences, Faculty of Biology, Medicine and Health, University of Manchester, Manchester, United Kingdom; ^3^Medical Physics University Hospital Southampton NHS Foundation Trust, Southampton, United Kingdom; ^4^Clinical Experimental Sciences, University of Southampton, Southampton General Hospital, Southampton, United Kingdom; ^5^Centre for Preventive Neurology, Wolfson Institute of Population Health, Queen Mary's University of London, London, United Kingdom; ^6^Department of Basic and Clinical Neuroscience, Institute of Psychiatry, Psychology, and Neuroscience, King's College London, London, United Kingdom

**Keywords:** Alzheimer's disease, Aβ PET, plasma biomarkers, machine learning classification algorithm, feature selection, feature matching

## Abstract

**Background:**

Alzheimer's disease (AD) greatly affects the daily functioning and life quality of patients and is prevalent in the elderly population. Amyloid-β (Aβ) accumulation in the brain is the main hallmark of AD pathophysiology. Positron Emission Tomography (PET) imaging is the most accurate method to identify Aβ deposits in the brain, but it is expensive and not widely available. The development of a low-cost method to detect Aβ deposition in the brain, as an alternative to PET, would therefore be of great value. This study aims to develop and validate machine learning algorithms for accurately predicting brain Aβ positivity using plasma biomarkers, genetic information, and clinical data as a cost-effective alternative to PET imaging.

**Methods:**

We analyzed 1,043 patients from the Alzheimer's Disease Neuroimaging Initiative (ADNI) dataset and validated our models on 127 patients from the Center for Neurodegeneration and Translational Neuroscience (CNTN) dataset. Brain Aβ status was determined using plasma biomarkers [Aβ42, Aβ40, Phosphorylated tau (pTau) 181, Neurofilament light chain (NfL)], Apolipoprotein E (APOE) genotype, and clinical information [Mini-Mental State Examination (MMSE), Montreal Cognitive Assessment (MoCA), age, education year, and gender]. Decision tree, random forest, support vector machine, and multilayer perceptron machine learning methods were used to combine all this information. We introduced a feature selection method to balance the performance and the number of features. We conducted a feature matching technique to enable our model to be tested on the external dataset without retraining.

**Results:**

Our system achieved a value of 0.95 for the Area Under the ROC curve (AUC) using the ADNI dataset (*n* = 340) and the full set of 11 features. Our architecture was also tested on an external dataset (CNTN, *n* = 127) and achieved an AUC of 0.90. When using only five features (pTau 181, Aβ42/40, Aβ42, APOE ɛ4 count, and MMSE) on 341 ADNI patients, we achieved an AUC of 0.87.

**Conclusion:**

The random forest, support vector machine and multilayer perceptron methods can accurately predict brain Aβ status using plasma biomarkers, genotype, and clinical information. The method generalizes well to an independent dataset and can be reduced to using only five features without losing much accuracy, thus providing an inexpensive alternative to PET imaging.

## 1 Introduction

Alzheimer's Disease (AD) is the most common form of dementia that mostly happens in those aged 65 or above (NIH, 2023). According to the World Health Organization (WHO), more than 55 million people are living with dementia around the world in 2023, and 60-70% of them are AD patients (WHO, 2023).

The accumulation of amyloid-β (Aβ) and tau neurofibrillary tangles are the two main pathological hallmarks of AD (Radiological Society of North America, 2023). Aβ is a peptide originating from the Amyloid Precursor Protein (Dementias Platform UK, 2021). It is found most commonly in two forms, Aβ40 and Aβ42, with the longer form being more toxic. In the brains of AD patients, Aβ cannot be cleared effectively, which leads to the accumulation of amyloid oligomers and plaques. Amyloid deposits inhibit synaptic function and ultimately kill neurons, predominantly in the hippocampus. Tau is a protein normally bound to microtubules in the axons, which play a role in transporting messages between neurons. For patients with AD, their tau proteins leave the microtubules to form neurofibrillary tangles, damaging neuronal structure and function.

Although there is currently no cure for AD (NIH, 2023), amyloid-clearing therapies (most recently antibodies that target Aβ) can slow down the progress of the disease and improve the quality of life for patients in the first stages of the disease. This new generation of drugs is likely to be most effective when given as early as possible, ideally before any disease symptoms are evident. An early diagnosis and prognosis are therefore crucial for potential patients to receive timely treatments. The key to diagnosis is the accurate detection of Aβ deposits.

Positron Emission Tomography (PET) imaging is currently the state-of-the-art method to diagnose AD. Using imaging agents that can bind to Aβ deposits, such as^11^C-labeled Pittsburgh compound B (PIB), PET can clearly detect and quantify Aβ accumulation in the brain. However, PET imaging is expensive, the radioactive tracer is unsuitable for patients with specific health conditions, and few hospitals are equipped with PET scanners. There is, therefore, an urgent need to develop a low-cost and easily accessible method for the diagnosis of AD that can substitute for PET imaging.

Plasma blood biomarkers can be collected easily and are much cheaper than PET imaging. Antibody-based methods, such as ELISA, electrochemiluminescence, and Simoa, are typically used. The presence of specific plasma biomarkers has been found to be correlated with Aβ deposition in the brain. Therefore, estimating the brain Aβ status may be possible using the plasma biomarkers.

Various machine learning architectures have been proposed for the diagnosis of AD using plasma biomarkers. Pan et al. (2023) proposed a decision tree (DT) classification algorithm to predict the Aβ status using plasma biomarkers and cognitive test results. They enrolled 609 patients from hospitals and extracted 14 features from the patients as their dataset. They prepared three models with different numbers of features on their study cohort. Their DT model gave an Area Under the ROC curve (AUC) value of 0.94 on the dataset with 14 features, 0.83 on the dataset with 5 features, and 0.71 on the dataset with 3 features. Vergallo et al. (2019) introduced a method to predict the brain Aβ status using the plasma Aβ40/42 ratio in cognitively normal individuals. They collected a dataset from the INSIGHT-preAD study (Dubois et al., 2018). They identified the ratio of Aβ40/42 as the most relevant feature for the Aβ prediction by the random forest (RF) and classification-and-regression-trees algorithms. They showed the Aβ40/42 ratio was able to estimate the brain Aβ status with 0.79 AUC. Youn et al. (2022b) developed machine learning algorithms to estimate the brain Aβ PET positivity using plasma Aβ. Their dataset was from the Alzheimer's Disease All Markers Study (Youn et al., 2022a). They developed RF, support vector machine (SVM), logistic regression, and deep neural network algorithms using features of blood Aβ levels, age, Apolipoprotein E (APOE) genotype, and Mini-Mental State Examination (MMSE) scores. The RF achieved the best performance with 0.77 accuracy. Yang et al. (2023) used a stepwise logistic regression model to predict the positive Aβ PET with the plasma biomarkers. They collected the dataset from the Center for Neurodegeneration and Translational Neuroscience (CNTN) data center (CNTN, 2015). Their model estimated the Aβ PET status using Glial fibrillary acidic protein (GFAP) and Phosphorylated tau (pTau) 181 with 0.86 AUC in all patients (57 cognitively unimpaired and 87 cognitively impaired) and 0.93 AUC in cognitively impaired patients. Moradi et al. (2024) proposed a machine learning model to estimate the Aβ status based on demographics, APOE genotype, Magnetic Resonance Imaging (MRI), and neuropsychological assessments. The status of Aβ was defined by PET and Cerebrospinal Fluid (CSF) measurements. Their dataset was acquired from the Alzheimer's Disease Neuroimaging Initiative (ADNI) database (ADNI, 2004). They developed the ridge logistic regression (RLR) model and achieved a 0.68 AUC score in status estimation of Aβ PET. Ashton et al. (2019) created an Aβ positivity classification model with plasma biomarkers. They acquired the dataset from the Australian Imaging, Biomarker and Lifestyle Flagship Study of Aging (AIBL) (AIBL, 2006) for their study. They developed an SVM algorithm to predict the amyloid burden positivity with a different number of features. Their models gave an AUC of 0.891, using 12 features [Prothrombin, Adhesion GPCR F4, Aβ A4 protein, NGN2, APOE ɛ4 count, DNAH10 (axonemal), REST, Neurofilament light chain (NfL), RPS6KA3, GPSM2, FHAD1 and age] from the cognitively unimpaired cohort, 0.904 AUC using 10 features (APOE ɛ4 count, Aβ A4 protein, NfL, NGN2, DNAH10 (axonemal), REST, APBB3, GPSM2, Prothrombin, and FHAD1) from the Mild Cognitive Impairment (MCI) and AD cohort, and 0.725 AUC using only demographic features (gender, age, and APOE ɛ4 count) in the cognitively unimpaired cohort. Ko et al. (2019) developed a brain Aβ positivity prediction model with patients' demographic information, APOE genotype, and neuropsychological test results. They used the ADNI dataset as their study dataset. They introduced an adaptive Least Absolute Shrinkage and Selection Operator algorithm to identify the highly relevant features to the Aβ PET status. Their model achieved 0.754 AUC in the mild change cohort (cognitively normal, significant memory concern, and early MCI), 0.803 in the moderate change cohort (significant memory concern, early MCI, and late MCI), and 0.864 in severe change cohort (early MCI, late MCI, and AD). Ten Kate et al. (2018) proposed an estimation system to predict positive Aβ using non-invasive features, such as demographic information, cognitive data, and APOE genotype of the patients. Their study cohort was from the NeuGrid platform (NEUGRID4YOU, 2015). Their SVM model gave prediction results of 0.81 AUC in MCI and 0.74 AUC in cognitively normal patients.

Previous studies have thus demonstrated the feasibility and clinical utility of estimating brain Aβ PET status using plasma biomarkers, APOE genotype, and clinical information. The field has matured significantly, with multiple studies achieving AUC values above 0.90 and commercial assays receiving regulatory approval for clinical use. Various machine learning algorithms, such as DT and SVM, have been developed and shown to perform well in predicting Aβ PET status. These findings provide a strong foundation for our study.

However, several challenges remain in translating these promising results to broader clinical practice. Existing studies primarily emphasize achieving high accuracy within single-cohort settings, often overlooking practical constraints related to feature quantity, computational efficiency, and model generalizability across different datasets and populations. Most published models require retraining when applied to new datasets or when key features are unavailable, limiting their practical utility. Additionally, there remains a need for systematic comparison of multiple machine learning approaches under standardized conditions and validation across independent external datasets.

To address these practical challenges, we propose a comprehensive machine learning framework that incorporates feature selection methods to maintain high accuracy with minimal features, and feature matching techniques that enable external dataset testing without model retraining. Our approach emphasizes model robustness and generalizability, critical factors for real-world clinical implementation that have received limited attention in previous studies.

Our system achieved a 0.95 AUC value to estimate the amyloid PET positivity in the ADNI dataset, which is competitive with existing approaches, and also achieved a high AUC of 0.90 when independently tested on the CNTN dataset. Building upon the established foundation of plasma biomarker research and commercial implementations, we developed four distinct machine learning classification algorithms with a focus on practical deployment challenges, including model generalizability without retraining and computational efficiency. Our specific contributions include systematic external validation and the development of methods to maintain performance with reduced feature sets, addressing key gaps in the translation from research to clinical practice.

## 2 Materials and methods

### 2.1 ADNI and CNTN

The ADNI database, a public dataset especially for AD research, contains various types of data, such as patient clinical information, biomarker data, and medical test results, making it suitable for this research target.

Another dataset is required to verify the robustness and generalization ability of the machine learning algorithms. The CNTN data center, committed to studying neurodegenerative diseases in the aging population, such as Alzheimer's and Parkinson's, is an ideal test dataset.

The data used in this study were obtained from the ADNI database (adni.loni.usc.edu) and CNTN data center (nevadacntn.org). The ADNI and CNTN studies were conducted with informed consent from all participants or their authorized representatives, and the study protocols were approved by the institutional review boards of all participating institutions.

### 2.2 Study cohort

In the ADNI dataset, 1,043 patients were included in this study. We prepared three datasets with different groups of features for different purposes as follows:

**The full feature dataset** with the most features was used to develop the four machine learning algorithms and tune the hyperparameters.

**The best feature dataset** with fewer features was designed to optimize the trade-off between performance and the number of features.

**The trimmed feature dataset** with the same features as the CNTN dataset was used to test the generalization ability of the algorithms.

Table 1 indicates the details of each dataset used in this research project. The first three datasets are from the ADNI database by selecting different groups of features. There are 340 patients with 11 features that can be found in the ADNI database as the full feature dataset, 341 patients with the 5 features as the best feature dataset, and 1,043 patients with the 8 features as the trimmed feature dataset.

**Table 1:** Study cohort information.

**Source**	**ADNI**			**CNTN**
**Dataset**	**Full feature**	**Best feature**	**Trimmed feature**	**External dataset**
Patients	340	341	1043	127
Features	pTau181	pTau181	pTau181	pTau181
	APOE4	Aβ42/40	APOE4	APOE4
	NfL	Aβ42	NfL	NfL
	Aβ42/40	MMSE	MoCA	MoCA
	Aβ42	APOE4	MMSE	MMSE
	Aβ40		Age	Age
	MoCA		Education	Education
	MMSE		Gender	Gender
	Age			
	Education			
	Gender			

The features used in this study are as follows:

- Plasma biomarkers: pTau 181 is the tau protein with Ser181 phosphorylated. Tau hyperphosphorylation is common in AD (Shen et al., 2021; Mattsson-Carlgren et al., 2021; Therriault et al., 2023). The higher pTau 181 level is correlated to Aβ positivity. Aβ42 and Aβ40 are the most common forms of Aβ. Aβ42 is more prone to aggregation, while Aβ40 is relatively stable (Cheng et al., 2022). When the Aβ42 accumulates in deposits in the brain, the concentration of Aβ42 in the plasma decreases, which leads to a lower Aβ42/40 ratio in the plasma (Wisch et al., 2023). NfL forms part of the neurofilament within large-caliber myelinated axons. When axons are damaged or neurons degenerate, NfL levels increase and are released into the blood (Rauchmann et al., 2021). A higher plasma NfL concentration is related to a severe brain Aβ burden.
- There are three main APOE genotypes: APOE ɛ2, ɛ3, and ɛ4. The APOE ɛ4 genotype is a significant genetic risk factor for AD (Weigand et al., 2021). Being homozygous for APOE ɛ4 has a higher risk for AD than being heterozygous. The number of APOE ɛ4 was counted as the feature in this study.
- Demographic information: age, gender, and years of education.
- Neuropsychological tests: The MMSE test includes 30 questions covering language, memory, attention, reading, and writing ability. The total score range is from 0 to 30. Patients with lower scores are more likely to be at risk of cognitive impairment. The Montreal Cognitive Assessment (MoCA) test also includes 30 questions but is more complex than the MMSE. MoCA includes a visuospatial test component. MoCA is more sensitive to the early stage of cognitive impairment.

The plasma biomarkers, APOE genotype, and clinical information data were downloaded from the ADNI database (“University of Gothenburg Longitudinal Plasma P-tau181 [ADNI1, GO, 2] Version 2020-06-18.csv,” “ADNIMERGE - Key ADNI tables merged into one table [ADNI1, GO, 2, 3].csv,” and “Blennow Lab ADNI1-2 Plasma neurofilament light (NFL) longitudinal [ADNI1, GO, 2] Version 2018-10-03.csv”).

### 2.3 Feature selection

For the full feature dataset, we used features known to be relevant to AD.

For the best feature dataset, we calculated the importance scores of the features from the full feature dataset using random forest, which achieved the highest AUC value among the decision tree (DT), random forest (RF), support vector machine (SVM), and multilayer perceptron (MLP) algorithms (Results Section 4.1).

**Figure 1:**
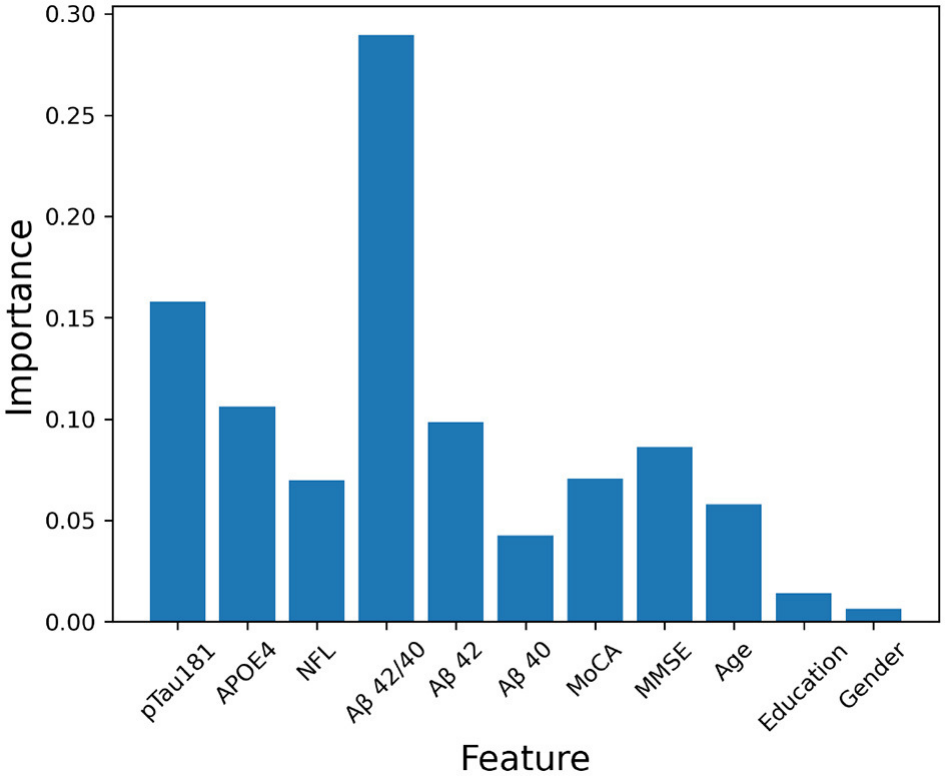
Feature importance scores.

During the training process, RF evaluates the importance of each feature by measuring its contribution to the Gini impurity reduction when it is used to split the dataset. The importance score of each feature can be calculated by averaging the decrease in Gini impurity caused by this feature across all trees in the forest. The feature with the higher importance score is considered the more important, indicating a stronger contribution to the model's predictive power. The importance score of each feature is shown in Figure 1. For a fair comparison, we selected five features for our best feature dataset, the same feature amount as the best model of the state-of-the-art work (Pan et al., 2023). The five features with the highest importance scores were selected for the best feature dataset. The features were pTau 181, Aβ42/40, Aβ42, APOE ɛ4 count, and MMSE.

The features were used in the trimmed feature dataset to match those in the CNTN dataset, as the CNTN dataset lacks some information compared to the full feature dataset.

### 2.4 Feature matching

To enable direct testing of our model on the external dataset, we selected the same group of features for the trimmed feature dataset as those used in the CNTN dataset. Since the CNTN dataset and the ADNI trimmed feature dataset originate from different data sources, we applied z-score standardization to both datasets, ensuring consistency in feature value range and distribution. We also utilized z-score standardization for the remaining datasets to eliminate the impact of feature scale differences on the model performance.

### 2.5 Amyloid β PET status

ADNI database provided processed labels for the Aβ PET status, 0 for negative and 1 for positive.

The Aβ PET status information data was downloaded from the ADNI database (“UC Berkeley - amyloid PET 6mm Res analysis [ADNI1, GO, 2, 3, 4].csv”).

### 2.6 Raw data preprocessing

The data collected from the ADNI and CNTN databases are distributed in different files and formats. To make the data suitable for machine learning algorithms, the collected data needs to be preprocessed. The steps of data preprocessing are as follows:

1. Locate the label (Aβ PET status) and features (each plasma biomarker test result, APOE genotype, and clinical information) data in corresponding data files.
2. Unify the format of the sampling date.
3. Extract sampling results and corresponding sampling date for the label and each feature.
4. Combine the label with all required features into the complete samples. Only keep the samples with all the features sampled within 90 days before or after the label sampled date.
5. Transfer categorical features into numbers and standardize the continuous value features with the z-score standardization method.

### 2.7 8-fold cross validation

**Figure 2:**
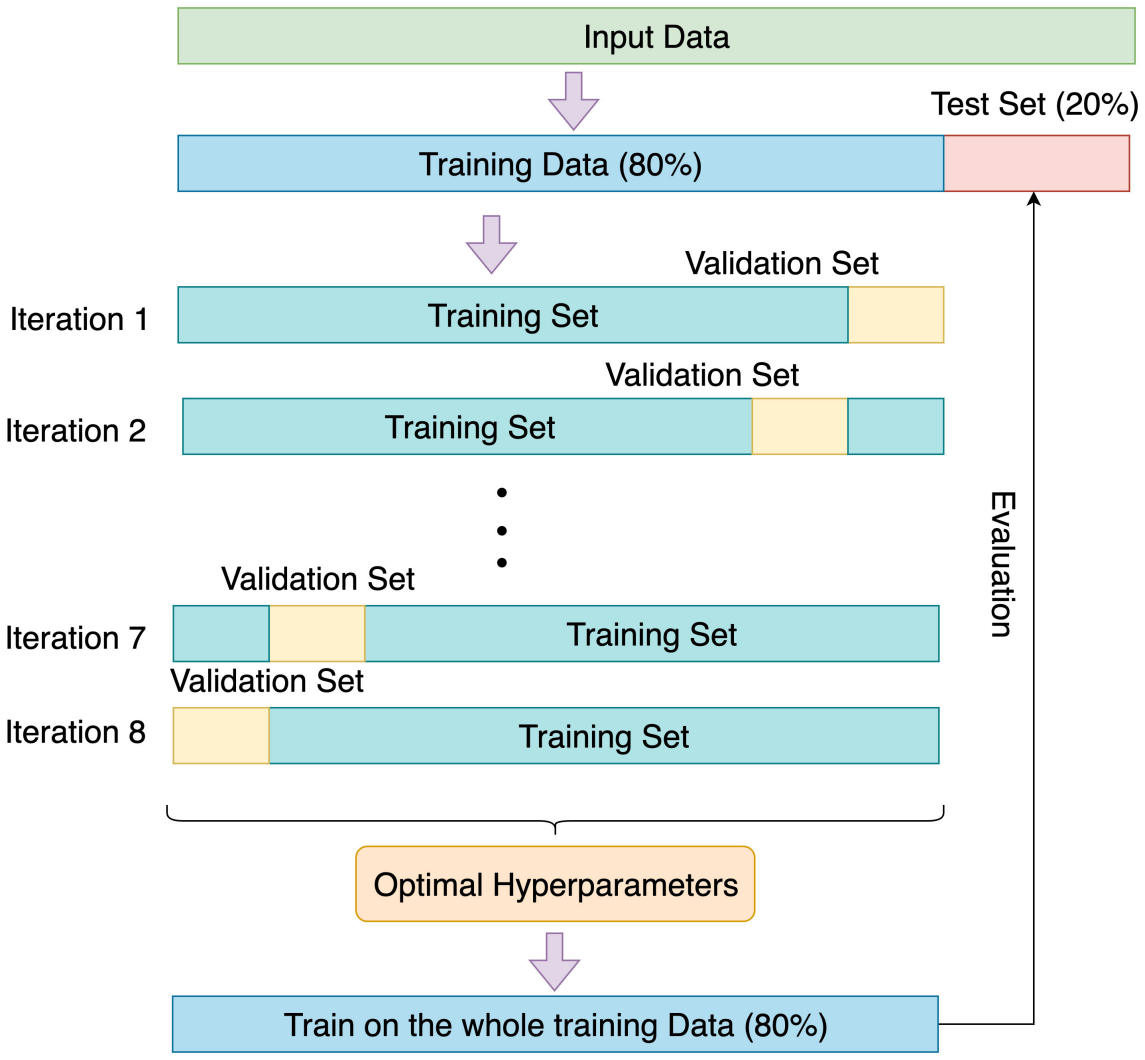
8-fold cross validation.

The 8-fold cross validation was conducted to tune the hyperparameters and test the models. Figure 2 shows the process of 8-fold cross validation.

20% of the patients were randomly picked as the test set, and the remaining 80% of the patients were split into 8 equal-sized groups. Each group was used as the validation set once, and the remaining 7 groups were pooled to be used as the training set. The hyperparameters were tuned to optimize the performance of the 8 validation sets. Finally, the entire training data (80% patients) was used to train the model with the optimal hyperparameters, and the model was tested on the test set (20% patients) to evaluate the performance.

## 3 Machine learning algorithm design

### 3.1 Rationale for algorithm selection

We selected four machine learning algorithms, DT, RF, SVM, and MLP, which are widely used and achieved good performance in related works. The architectures of these algorithms have good interpretability, and the characteristics of these algorithms are very suitable for our research as follows.

DT is straightforwardly interpretable because its structure can be visualized to explain the classification process. Since it is widely used in many related works and performs well, it was considered in our study.

RF is an ensemble learning method consisting of multiple DTs. By combining the results of multiple DTs, the ensemble method can achieve better performance than a single tree.

SVM is a robust classification algorithm capable of addressing both linear and non-linear problems. It is particularly effective in handling high-dimensional data and is well-suited for classification tasks involving a large number of features. In this study, we chose the SVM algorithm due to its strong performance on small to medium-sized non-linear datasets.

MLP is the most basic neural network with a good ability for generalization. The MLP was chosen for this study due to the medium size of the dataset, its ability to handle non-linear data, and the ease of implementing and adjusting the MLP's network structure.

### 3.2 DT

#### 3.2.1 Structure of DT

**Figure 3:**
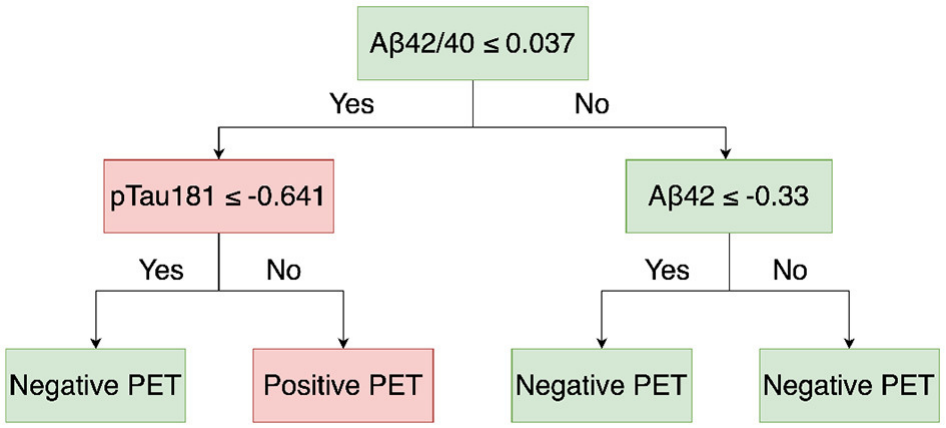
Demonstration of DT structure.

Figure 3 shows a demonstration of DT structure. The tree was built from a root node, and all the training data were included. Then, the node was split into two child nodes following the condition of the feature, which minimized the Gini impurity. Although the right child tree did not distinguish the classes, the Gini impurity was reduced by the condition. The whole tree was constructed by recursively splitting the node until the stop conditions (the maximum depth, the minimum sample split, and the minimum sample leaf) were reached.

#### 3.2.2 Hyperparameter tuning of DT

The grid search technique was used to tune the hyperparameters of the DT. Grid search is a hyperparameter tuning method (Anggoro and Mukti, 2021), which can find the hyperparameter combination in the given grid with the best score in a specific performance metric (Géron, 2022). Table 2 shows the hyperparameters tuning setup for the DT. Max depth limits the maximum depth of the tree. Min samples split specifies the minimum number of samples required to split an internal node. Min samples leaf sets the minimum number of samples required to be a leaf node.

**Table 2:** Grid search setting of DT.

Max depth	4, 5, 6, 7, 8, 9, 10, 11, 12, 13, 14, 15, 16
Min samples split	4, 5, 6, 7, 8, 9, 10, 11, 12, 13, 14, 15, 16
Min samples leaf	2, 3, 4, 5, 6, 7, 8, 9, 10

According to the grid search, the optimum combination of the hyperparameters is the maximum depth of 4, the minimum sample split of 11, and the minimum sample leaf of 2.

### 3.3 RF

#### 3.3.1 Diversity of RF

The RF is an ensemble architecture that consists of multiple DTs. In order to achieve better performance, the core idea of the ensemble method is to make each individual tree different from each other. One method that can maximize the diversity of the individuals is random feature selection, which randomly selects a subset of features for each individual tree.

#### 3.3.2 Hyperparameter tuning of RF

Since the RF is based on the DT, the hyperparameters include the tree and ensemble hyperparameters. The tree hyperparameters are reused from the DT optimized from the previous Section 3.2.2, with a maximum depth of 4, a minimum sample split of 11, and a minimum sample leaf of 2. The ensemble hyperparameters are the number of trees and the maximum features. Table 3 shows the grid search setting.

**Table 3:** Grid search setting of RF.

Number of trees	30, 50, 100
Max features	2, 3, 4

The optimum ensemble hyperparameters of the RF model were found to be a number of trees of 100 and the maximum features of 2.

### 3.4 SVM

#### 3.4.1 Kernel selection

The kernel function is the core of the SVM algorithm. The most commonly used kernel functions are linear, polynomial, and Gaussian (radial basis function) kernels. Three kernels were tested in this study.

The computational resource requirement for the linear kernel is the lowest. It can only handle linearly separable data. The linear kernel function is


(1) $$\begin{array}{l} K\left(x,x^{\prime }\right)=x^{T}x^{\prime } \end{array}$$

where *x, x*′ are the two distinct data points. Superscript T represents the transpose of the vector. *x*^*T*^*x*′ is the dot product of the data points.

Polynomial kernel and Gaussian kernel can be used to process non-linear separable data. Both map the data into a higher-dimensional space to realize linear separability. The difference between them is the mapping method.

The Gaussian kernel uses the Gaussian function to map the data into a higher dimensional space (Yang et al., 2021). The Gaussian kernel function is


(2) $$\begin{array}{l} K\left(x,x^{\prime }\right)=\exp\left(-\gamma ||x-x^{\prime }|{|}^{2}\right) \end{array}$$

where ɣ is the hyperparameter which controls the width of the Gaussian function. The larger ɣ narrows the Gaussian function. ||*x*−*x*′|| is the Euclidean distance between the data points.

The Gaussian kernel excels at processing data with local correlations because it calculates the distance between the data points.

The polynomial kernel uses the polynomial function to map the data into a higher dimensional space. The polynomial kernel function is


(3) $$\begin{array}{l} K\left(x,x^{\prime }\right)={\left(\lambda x^{T}x^{\prime }+r\right)}^{d} \end{array}$$

where λ is the hyperparameter that controls the scaling of the dot product, *r* is the hyperparameter that controls the bias, *d* is the degree of the polynomial, *x*^*T*^*x*′ is the dot product of the data points.

The polynomial kernel is well-suited for data with global correlations since it calculates the dot product of the data points.

#### 3.4.2 Hyperparameter tuning of SVM

The hyperparameters were tuned using a grid search. The grid setting was shown in Table 4. C is the regularization parameter. Too large C narrows the margin of SVM, which may lead to overfitting. Too small C widens the margin, which may lead to underfitting. The λ in the polynomial kernel by default is 1.0/number of features (Scikit-learn, 2023), which is adaptive for datasets with various numbers of features.

**Table 4:** Grid search setting of SVM.

Linear kernel	
C	0.1, 0.2, 0.5, 1, 5, 10, 20, 50, 100
**Gaussian kernel**	
ɣ	0.001, 0.01, 0.02, 0.05, 0.1, 0.5, 1, 2, 5, 10
C	0.1, 0.2, 0.5, 0.7, 1, 1.5, 2, 3, 5, 6, 7, 8, 9, 10
**Polynomial kernel**	
Degree, d	2, 3
r	0.1, 1, 10, 20, 50
C	0.1, 1, 2, 3, 5

According to the grid search, the optimal hyperparameters were found, the Gaussian kernel with the ɣ of 0.01 and the C of 10.

### 3.5 MLP

#### 3.5.1 Structure of MLP

**Figure 4:**
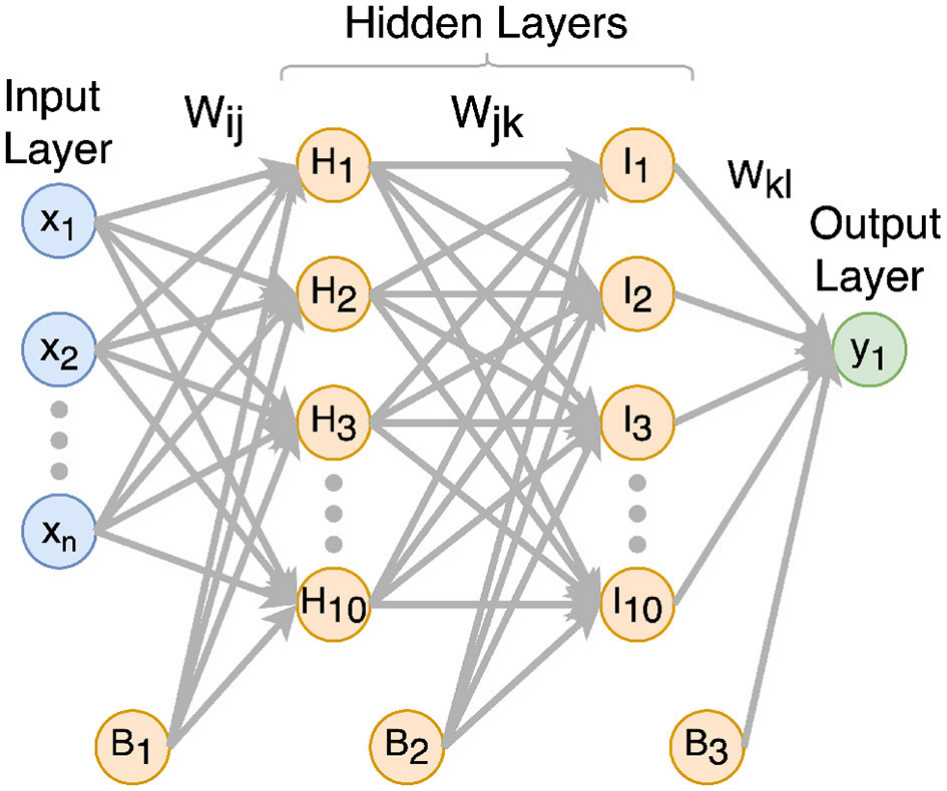
Structure of MLP.

The structure of the designed MLP algorithm is illustrated in Figure 4. There is one input layer with many neurons for feature input, two hidden layers with 10 neurons for each, and one neuron as the output layer for the estimation result. The MLP is a fully connected neural network, which means all the neurons in the previous layer are connected to all the neurons in the next layer. The output neuron presents the probability of the positive class calculated by a sigmoid function. If the probability is >0.5, the result is positive; otherwise, the result is negative.

#### 3.5.2 Hyperparameter tuning of MLP

The hyperparameter tuning is an essential part of implementing the MLP algorithm. The ReLU function (below) is infinitely differentiable, and its Equation 4 is concise for calculation (Eckle and Schmidt-Hieber, 2019). The ReLU function is the most widely used activation function in neural networks' hidden layers, and it usually performs very well.


(4) $$\begin{array}{l} f\left(x\right)=\max\left(0,x\right) \end{array}$$

The Adam optimizer can adaptively adjust the learning rate during the network training process (Zhang, 2018). The Adam optimizer was selected for the designed MLP algorithm because the Adam optimizer converged faster and was more robust than basic optimizers such as stochastic gradient descent (Kingma and Ba, 2015).

The remaining hyperparameters, such as hidden layer structure, batch size, dropout rate, and epochs, were tuned with the help of a grid search, as presented in Table 5. The hidden layer sets the number of neurons in each hidden layer. The dropout rate is the probability of the neurons to be dropped out to prevent overfitting. The epoch is the number of times the entire training set passed to the network. The batch size is the number of samples used in each iteration to update the weights.

**Table 5:** Grid search setting of MLP.

Hidden layer	(10, 10), (30, 10), (30, 30), (10, 10, 10), (30, 10, 10)
Dropout rate	0.2, 0.5, 0.7
Epoch	500, 750, 1000
Batch size	50, 100, 200, 400

The optimum hyperparameter combination for the MLP is a hidden layer structure of (10, 10), a dropout rate of 0.5, an epoch of 750, and a batch size of 50.

**Figure 5:**
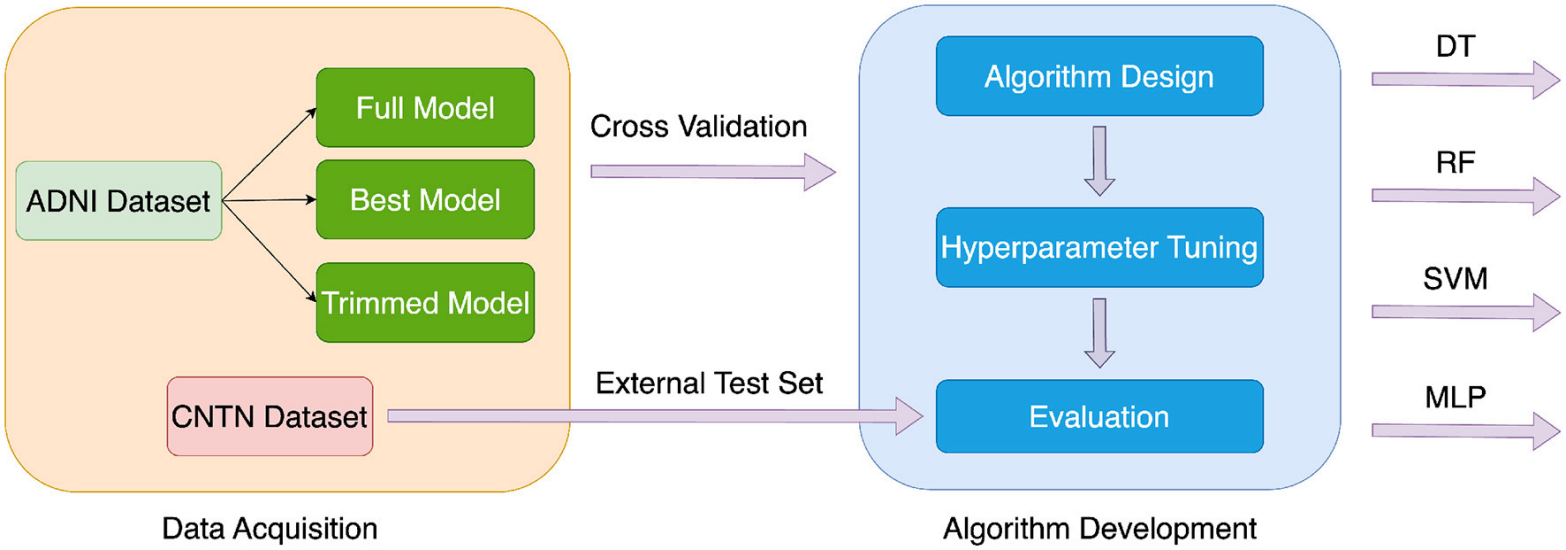
Entire workflow. In the data acquisition part, the data was collected from the ADNI database and CNTN dataset. The feature selection was conducted to prepare various datasets with different numbers of features. The data was preprocessed and ready to be used for the algorithm development part. In the algorithm development part, four machine learning algorithms were designed. The hyperparameters were fine-tuned. The various performance metrics were used to evaluate the comprehensive performance of each algorithm. The results of all the algorithms were compared. An external dataset was used to test the generalization ability and robustness of the model.

**Figure 6:**
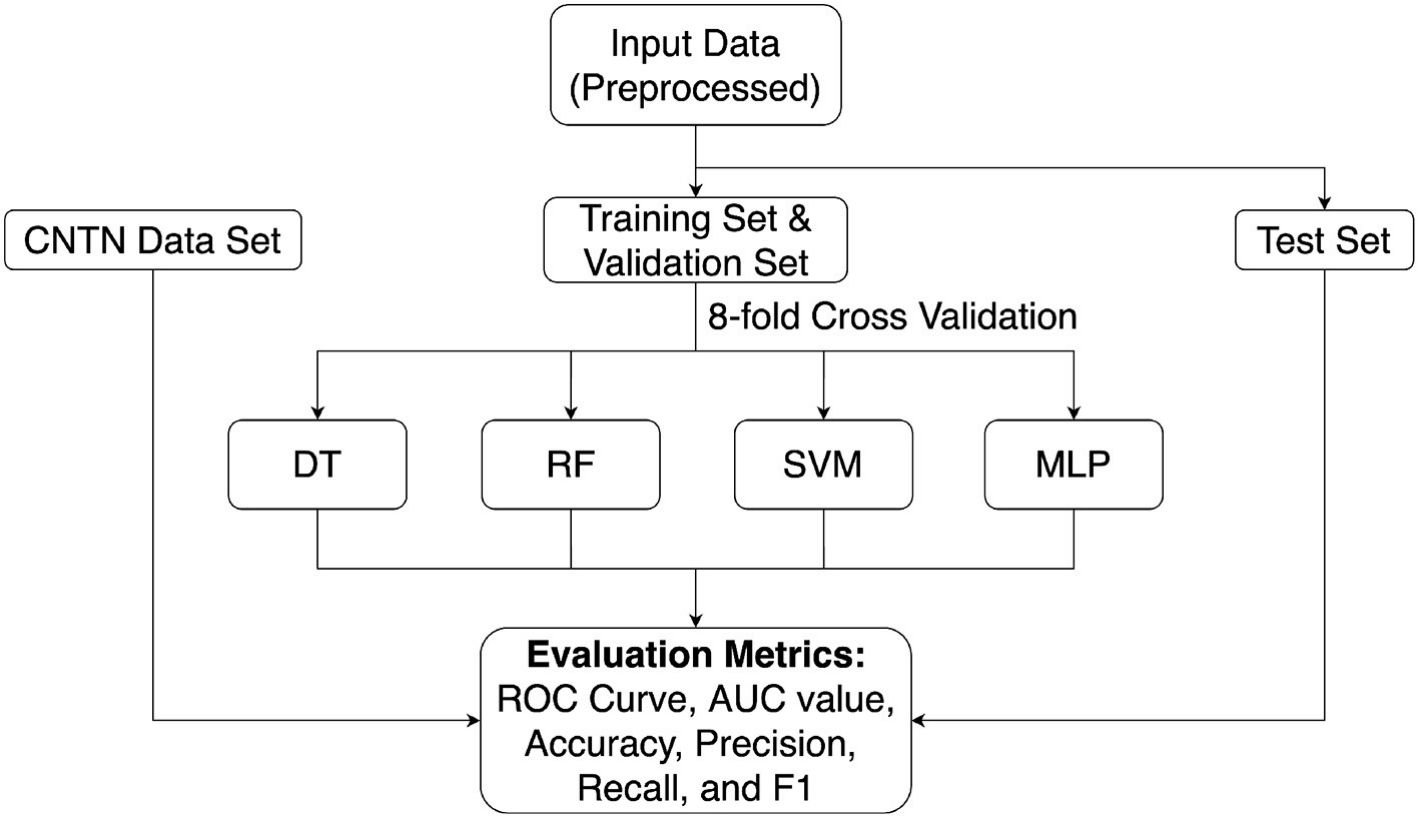
Machine learning framework. First, the preprocessed ADNI dataset was split into the training data and test set. The training set and validation set were split by 8-fold cross validation from training data. Then, the training and validation sets were used to train the model and help with hyperparameter tuning. The hyperparameters of each algorithm were tuned on the full feature dataset and were kept the same on the best feature dataset and the trimmed feature dataset. Finally, the model was evaluated on the test set. Various performance metrics such as ROC curve, AUC value, accuracy, precision, recall, and F1 score were calculated to evaluate the model performance. In addition, the CNTN dataset was used as an external test set.

The entire workflow of the system is shown in Figure 5. The framework of machine learning architecture implementation is shown in Figure 6.

## 4 Results

Multiple performance metrics, AUC, accuracy, precision, recall, and F1 score, were used to evaluate and compare the performance of the four machine learning architectures tested on the three ADNI datasets (the full feature dataset, the best feature dataset, and the trimmed feature dataset) and an external dataset (the CNTN dataset). AUC was used to evaluate the comprehensive performance of a model as it considers both the true positive rate and the false positive rate. The other four performance metrics were used to evaluate the model performance from different perspectives, and their formulas are as follows:


(5) $$\begin{array}{lll} \text{Accuracy} & = & \frac{TP+TN}{TP+TN+FP+FN} \end{array}$$

(6) $$\begin{array}{lll} \text{Precision} & = & \frac{TP}{TP+FP} \end{array}$$

(7) $$\begin{array}{lll} \text{Recall} & = & \frac{TP}{TP+FN} \end{array}$$

(8) $$\begin{array}{lll} \text{F1} & = & 2\cdot \frac{Precision\cdot Recall}{Precision+Recall} \end{array}$$

### 4.1 Result of full feature dataset

The performance metrics outcomes for four algorithms applied to the full feature dataset are presented in Table 6. The RF achieved the highest scores in all performance metrics. MLP achieved higher scores in AUC and precision and lower scores in accuracy, recall, and F1 than the SVM. DT has the lowest scores in all performance metrics except for recall.

**Table 6:** Performance metrics on each dataset.

	**AUC**	**Accuracy**	**Precision**	**Recall**	**F1**
**Full feature dataset**					
DT	0.831	0.779	0.769	0.690	0.727
RF	0.951	0.897	0.958	0.793	0.868
SVM	0.918	0.824	0.815	0.759	0.786
MLP	0.938	0.794	0.826	0.655	0.731
**Best feature dataset**					
DT	0.776	0.765	0.811	0.769	0.789
RF	0.863	0.794	0.879	0.744	0.806
SVM	0.864	0.809	0.882	0.769	0.822
MLP	0.870	0.824	0.886	0.795	0.838
**Trimmed feature dataset**					
DT	0.792	0.716	0.686	0.735	0.709
RF	0.791	0.712	0.707	0.663	0.684
SVM	0.797	0.736	0.731	0.694	0.712
MLP	0.806	0.707	0.713	0.633	0.670
**CNTN dataset**					
DT	0.677	0.504	1.0	0.074	0.137
RF	0.886	0.661	0.963	0.382	0.547
SVM	0.886	0.787	0.936	0.647	0.765
MLP	0.896	0.787	0.936	0.647	0.765

**Figure 7:**
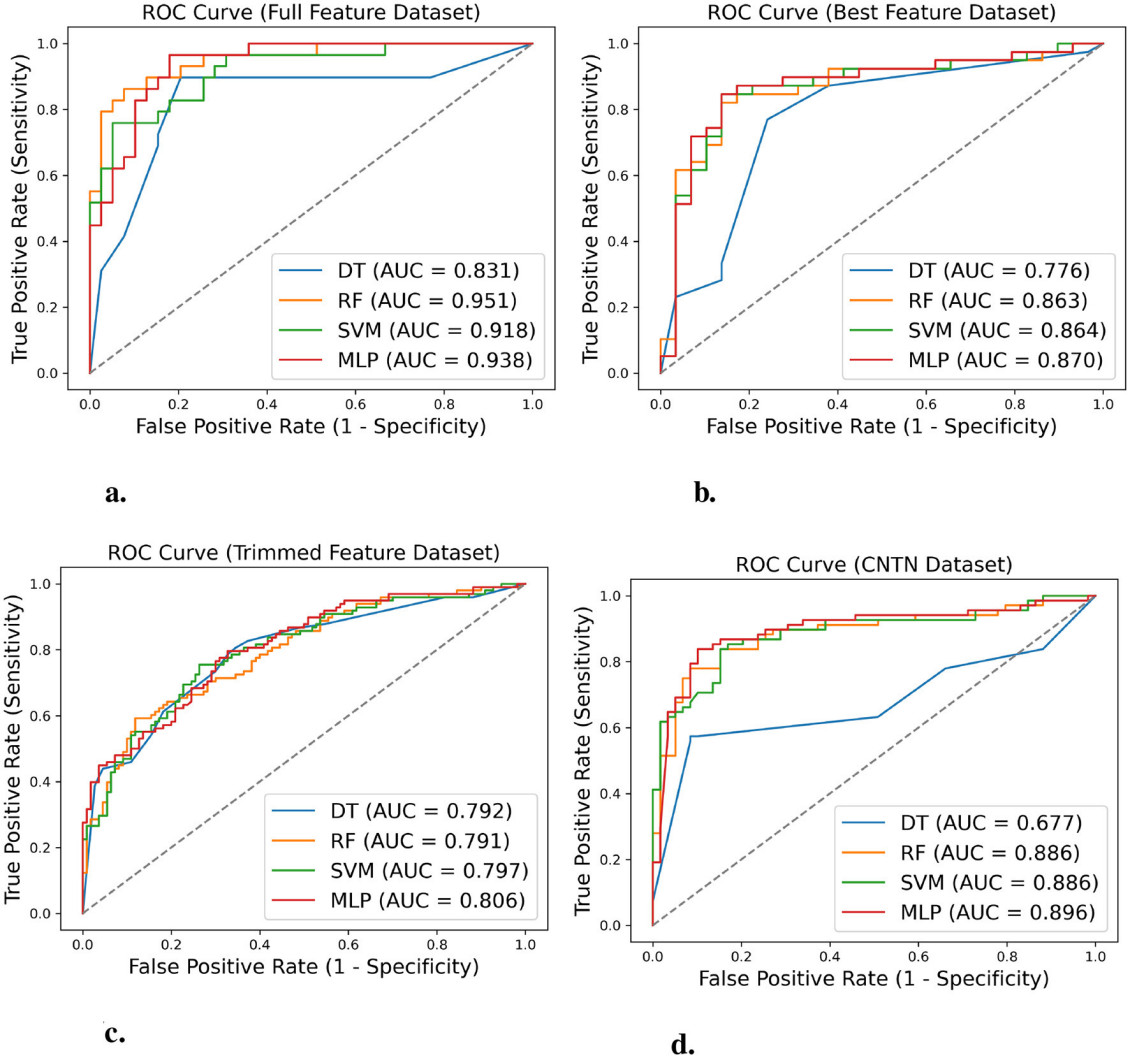
ROC curves comparing machine learning algorithm performance across different feature sets and datasets. ROC curves show the trade-off between true positive rate (sensitivity) and false positive rate (1-specificity) for decision tree (DT), random forest (RF), support vector machine (SVM), and multilayer perceptron (MLP) algorithms. **(a)** Performance on full feature dataset (*n* = 340, 11 features) with RF achieving the highest AUC (0.95). **(b)** Performance on best feature dataset (*n* = 341, 5 features) with MLP achieving the highest AUC (0.87). **(c)** Performance on trimmed feature dataset (*n* = 1,043, 8 features) showing similar performance across all algorithms. **(d)** External validation on CNTN dataset (*n* = 127, 8 features) demonstrating model generalizability with MLP achieving AUC of 0.90.

Figure 7a illustrates the Receiver Operating Characteristic (ROC) curve comparison for each algorithm on the full feature dataset. The curve represents the relationship between the true positive rate and the false positive rate when the threshold changes. The RF, SVM, and MLP performed better than the DT on this dataset.

### 4.2 Result of best feature dataset

The results of the performance metrics using four algorithms on the best feature dataset are illustrated in Table 6. MLP achieved the highest scores in all performance metrics. SVM has a close AUC score to RF and higher accuracy, precision, recall, and F1 than RF. Except for recall, DT got the lowest scores in the remaining performance metrics.

The ROC curves of the four algorithms, tested on the best feature dataset, are compared in Figure 7b. The curves demonstrate that the DT substantially underperformed the other algorithms on this dataset.

### 4.3 Result of trimmed feature dataset

The performance metrics for the four algorithms tested on the trimmed feature dataset are displayed in Table 6. The MLP achieved the highest AUC, the SVM achieved the highest accuracy, precision and F1, and DT achieved highest recall. All four algorithms had closely similar performances on this dataset with this set of features.

In Figure 7c, the comparison of the ROC curve for each algorithm on the trimmed feature dataset is displayed. The four curves are close to each other, indicating that the four algorithms performed similarly on this dataset.

### 4.4 Result of CNTN dataset

The CNTN dataset was tested with the four algorithms trained on the entire trimmed feature dataset.

The performance metrics of four algorithms on the CNTN dataset are summarized in Table 6. MLP reached the highest AUC. SVM and MLP achieved the same scores in the other four performance metrics, which means they gave the same prediction results and achieved the highest accuracy, recall, and F1. RF achieved the same AUC as SVM and the highest precision but lower recall and F1. DT performed in an unbalanced way with a precision of 1.0 but very low recall and F1.

Figure 7d presents a comparison of the ROC curves for all algorithms on the CNTN dataset. The DT performed much worse than the other algorithms on this dataset.

### 4.5 Comparison of architectures

Table 7 compares the AUC performance of each machine learning architecture.

**Table 7:** Performance comparison on AUC.

	**Full feature dataset**	**Best feature dataset**	**Trimmed feature dataset**	**CNTN dataset**
DT	0.831	0.776	0.792	0.677
RF	0.951	0.863	0.791	0.886
SVM	0.918	0.864	0.797	0.886
MLP	0.938	0.870	0.806	0.896

The RF model achieved the highest AUC value on the full feature dataset, and the MLP model achieved slightly higher AUC values on the remaining three datasets. The DT's overall performance is inferior to that of the RF, SVM, and MLP.

Table 8 compares our work with recent studies on estimating Aβ PET using plasma biomarkers on the whole cohort with the AUC values reported. Our study achieves an AUC of 0.95 using a random forest model with 11 features, which is competitive with the established literature including landmark studies by (Pan et al. 2023) and (Nakamura et al. 2018) that demonstrated AUCs exceeding 0.90. Our best feature model, using a MLP with 5 features, achieves an AUC of 0.87, which is competitive compared to the best feature models of other studies.

**Table 8:** Recent work of Amyloid β PET estimation with plasma biomarkers.

**References**	**Dataset size**	**Feature amount**	**Model**	**AUC**
Xu et al. (2025) (this article)	340	11 (full features)	Random forest	0.95
	341	5 (best features)	MLP	0.87
(Pan et al. 2023)	609	14 (full features)	Decision Tree	0.94
	609	5 (best features)	Decision Tree	0.83
(Palmqvist et al. 2019)	842	5	Logistic regression	0.87
(Nakamura et al. 2018)	373	2	Youden's index	0.91
(Vergallo et al. 2019)	276	1	ROC analysis	0.79
(Yang et al. 2023)	144	2	Stepwise logistic regression	0.86
(Moradi et al. 2024)	231	4	Ridge logistic regression	0.68
(Ashton et al. 2019)	169	10	SVM	0.90

## 5 Discussion

Four machine learning algorithms, DT, RF, SVM, and MLP, were selected for the Aβ PET positivity prediction. DT has high interpretability, and the tree structure of the decision rules can be visualized. RF is well known for robustness and can reduce overfitting by averaging multiple DTs. SVM often performs efficiently on not-too-large datasets. MLP is a neural network with a simple structure and good generalization ability. All these algorithms achieved previous success in biomarker-based models. The hyperparameters of the four machine learning architectures were optimized using the full feature dataset and subsequently reused for both the best feature dataset and the trimmed feature dataset. This approach was adopted to maintain consistent hyperparameters, thereby ensuring a fair comparison and enabling an assessment of the model's generalization ability across different feature sets. In the full feature dataset, the RF achieved the highest AUC value of 0.951, followed by the MLP with 0.938 and SVM with 0.918, while the DT model produced the lowest AUC value of 0.831.

Feature selection facilitates clinical feasibility. Identifying the important and dominant features can significantly reduce the detection costs and patients' body burden, and RF, with highly predictive accuracy and interpretability, is a feasible choice for selecting important features in clinical applications. The importance score of each individual feature was calculated according to the contribution to the Gini impurity reduction in the RF algorithm (Feature Selection Section 2.3). The AUC values for the RF and SVM models were very close, 0.863 and 0.864, respectively, while the MLP model displayed a slightly higher AUC of 0.870 on the best feature dataset. We balanced the trade-off between feature reduction and model performance. Despite reducing the number of features, the selected feature set demonstrated a high correlation with Aβ PET status. This dataset used significantly fewer features and preserved the robust performance. In clinical applications, the reduced feature group can also provide reliable prediction results. Clinicians can flexibly choose from the full feature group or the reduced feature group to satisfy the practical requirement of the highest accuracy or further cost-efficiency.

Many features are costly to measure in blood, particularly those that quantify the concentrations of proteins using antibodies. It is, therefore, of great value to remove any features that are expensive to collect and add little power to any prediction. A very high performance can be achieved using only five features, namely: pTau 181, Aβ42/40, Aβ42, APOE ɛ4 count, and MMSE. The APOE genotype and the MMSE test are cheap to measure, and only three antibodies are needed to measure pTau 181, Aβ40 and Aβ42 with an ELISA. Applying our method to patients is, therefore, straightforward and inexpensive. The finding that only five features provided high AUC has significant clinical and diagnostic implications, addressing the challenge of limited feature availability, making biomarker-based AD diagnosis more cost-effective and easier to implement in clinical settings.

The performance of the four algorithms on the trimmed feature dataset is not significantly different. The MLP model achieved an AUC of 0.806, 0.797 for SVM, 0.791 for RF, and 0.792 for DT. On the external dataset, the CNTN dataset was only used for an external test set and was not used to train our model. The hyperparameter tuning process only depends on the performance of the validation set of the ADNI dataset, as shown in Figure 6. Therefore, the overfitting issue can be prevented. The MLP model reaches its highest AUC of 0.896, while the SVM and RF follow closely with an AUC of 0.886 for both. This indicated that RF, SVM, and MLP effectively applied the available information in the trimmed dataset to test the CNTN dataset. However, the DT model achieved poor and unbalanced performance across all performance metrics on this dataset, indicating that the DT model had difficulty generalizing to the external dataset. The results on the CNTN dataset emphasize the effectiveness of the feature matching technique in enhancing the model's generalization ability to external datasets.

According to the results of four algorithms on each dataset, the RF model performed best on the full feature dataset, which is the main research target. The MLP achieved stable and high performance across all the datasets, exhibited powerful generalization ability, and excellent comprehensive predictive performance. The SVM showed a slightly lower performance than MLP in each dataset and also achieved a good generalization ability. The DT, the simplest model, performed poorest. Since DT is easy to overfit when handling high-dimensional data, the rigid decision boundaries of DT are not flexible enough to separate the complex data distributions. Instead, MLP and SVM have more flexible decision boundaries and more efficient overfitting prevention methods, such as regularization for MLP and margin maximization for SVM, enabling them to handle non-linear and high dimensional data well and have a better generalization ability. To address DT's overfitting problem, RF utilized the ensemble method by aggregating multiple DTs to achieve better performance and stability than a single DT. In real-world clinical practice, MLP and SVM can be applied to detect Aβ PET status for patients with various types and amounts of features. Although the generalization ability of RF was not as good as MLP and SVM, RF has the potential to be used to obtain the most accurate prediction in circumstances of patients with a large number of features.

Our study demonstrated the efficacy of feature selection and feature matching techniques. These techniques offer the potential to tackle the problem of feature amount constraints, reduce computational resource demands, and increase model generalization capability in practical applications. By comparing with existing approaches, our work used a smaller dataset and fewer features yet achieved competitive AUC values when compared to established methods in the field. Within the rapidly evolving landscape of plasma biomarker-based AD diagnosis, where commercial solutions such as PrecivityAD™, Elecsys pTau181, and Simoa-based platforms have already demonstrated clinical utility, our contribution lies in addressing specific methodological gaps related to model generalizability and practical deployment challenges. Hence, using plasma biomarkers as a low-cost alternative to PET is of established significance in clinical and diagnostic applications, and our work contributes to improving model robustness and addressing practical implementation challenges in diverse clinical settings.

### 5.1 Clinical applicability and translation

The clinical translation of our plasma biomarker-based pipeline presents both significant opportunities and practical challenges. From a clinical workflow perspective, our system offers several advantages over current diagnostic approaches. Our best model (pTau 181, Aβ42/40, Aβ42, APOE ɛ4 count, and MMSE) can be readily integrated into existing clinical practice, as APOE genotyping and MMSE testing are already standard procedures in many memory clinics. The plasma biomarker collection requires only a standard blood draw, making it accessible across diverse healthcare settings, including primary care facilities that lack specialized neuroimaging capabilities.

However, clinical implementation faces several hurdles. Current clinical decision-making relies heavily on imaging-based confirmation of Aβ pathology, and clinicians may require substantial evidence before accepting plasma biomarkers as reliable substitutes for PET imaging. The probabilistic nature of machine learning predictions must be carefully communicated to clinicians who are accustomed to more definitive diagnostic results.

The economic implications are substantial. With PET scans costing $3,000–$8,000 compared to $100–$1,250 for plasma biomarker panels (Pais et al., 2023), our approach could significantly reduce healthcare costs while enabling broader population screening. This cost-effectiveness is particularly relevant given the increasing focus on early AD detection and the growing availability of disease-modifying treatments that are most effective when administered early in the disease course.

Integration with existing diagnostic pipelines requires careful consideration. Our system is best positioned as a pre-screening tool rather than a standalone diagnostic method. In practice, patients with high-risk predictions could be prioritized for PET imaging, while those with low-risk scores might undergo continued monitoring or alternative diagnostic workups. This tiered approach maximizes the clinical utility of both plasma biomarkers and PET imaging while optimizing resource allocation.

### 5.2 Regulatory and implementation challenges

The regulatory pathway for clinical implementation presents complex challenges. Regulatory agencies such as the FDA and EMA require extensive clinical validation demonstrating not only analytical validity but also clinical utility and actionability. Our current validation, while promising, represents only the initial phase of the regulatory requirements. Large-scale, multi-site clinical trials will be necessary to demonstrate consistent performance across diverse populations and healthcare settings.

Data harmonization emerges as a critical challenge for widespread implementation. Our feature matching technique addresses some inter-dataset variability, but significant challenges remain in standardizing plasma biomarker measurements across different laboratories, analytical platforms, and patient populations. The observed performance difference between ADNI (AUC 0.95) and CNTN (AUC 0.90) datasets, while encouraging, highlights the importance of robust standardization protocols. Different laboratory techniques, storage conditions, and processing procedures can significantly impact biomarker measurements, potentially affecting model performance.

Patient diversity represents another significant regulatory challenge. The ADNI dataset, while valuable, predominantly includes well-educated, Caucasian participants from high-resource settings. Regulatory approval will require demonstration of model performance across diverse demographic groups, including underrepresented racial and ethnic minorities, varying socioeconomic backgrounds, and different healthcare systems. The potential for algorithmic bias in healthcare AI systems has become a major regulatory concern, necessitating comprehensive fairness assessments.

The international nature of healthcare requires consideration of varying regulatory frameworks. While the FDA's recent guidance on AI/ML-based medical devices provides some clarity, the European Union's Medical Device Regulation (MDR) and other international standards introduce additional complexity. Our system's requirement for periodic retraining or updating to maintain performance may necessitate continuous regulatory oversight rather than traditional one-time approval processes.

Quality assurance and clinical laboratory standards present additional implementation challenges. The Clinical Laboratory Improvement Amendments (CLIA) requirements in the US and similar international standards mandate rigorous quality control procedures for clinical laboratory tests. Implementing our machine learning pipeline within these regulatory frameworks requires careful attention to result reporting, quality metrics, and laboratory personnel training.

### 5.3 Interpretability and clinical decision-making

The interpretability challenge in clinical machine learning represents a fundamental tension between model performance and clinical acceptance. While our MLP model achieved the highest performance across datasets, its “black box” nature poses challenges for clinical implementation. Clinicians require understanding of how predictions are generated, both for clinical decision-making and for patient communication. The superior interpretability of our decision tree model, despite its lower performance.

**Figure 8:**
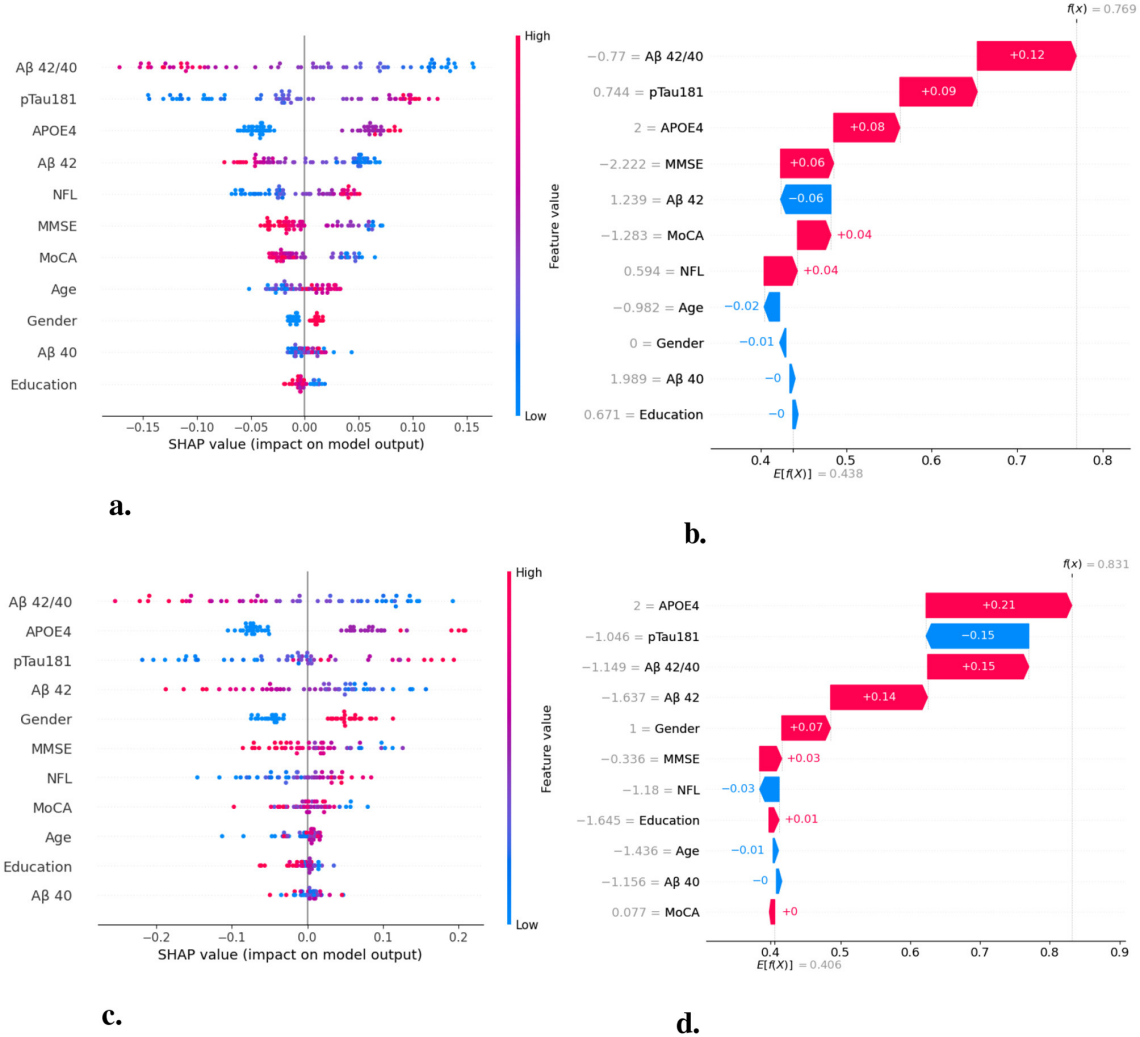
SHapley Additive exPlanations (SHAP) value analysis for model interpretability of random forest and multilayer perceptron algorithms. SHAP values quantify the contribution of each feature to individual predictions, providing both global feature importance and local explanations. **(a)** Beeswarm plot showing SHAP value distribution for RF model-each dot represents one patient, with color indicating feature value (red = high, blue = low) and x-axis position showing impact on prediction. **(b)** Waterfall plot for RF showing cumulative contribution of each feature to a single patient prediction, starting from baseline probability. **(c)** Beeswarm plot for MLP model showing similar feature importance patterns with Aβ42/40 ratio as the most influential predictor. **(d)** Waterfall plot for MLP demonstrating how individual feature values combine to produce final prediction probability for amyloid positivity.

Our random forest-based feature importance analysis provides some interpretability insights, identifying pTau 181 and Aβ42/40 ratio as the most predictive features. However, feature importance alone may not satisfy clinical interpretability requirements. Clinicians need to understand not just which features are important, but how specific feature values contribute to individual patient predictions. Figure 8 illustrates the use of SHAP (SHapley Additive exPlanations) values to provide global and local interpretability for our RF and MLP models. SHAP values quantify the contribution of each feature to the model's prediction, allowing clinicians to see how individual feature values influence the final risk score.

Patient communication represents another interpretability challenge. Patients and families require clear explanations of what Aβ positivity means, how the prediction was generated, and what the implications are for their care. The probabilistic nature of our predictions must be communicated in ways that patients can understand and act upon. This is particularly important given the emotional and psychological impact of AD-related diagnoses.

## 6 Conclusion

We developed an Aβ PET positivity estimation system utilizing cost-effective plasma biomarkers, genetic information, and clinical data. We devised a feature selection method to reduce the number of features while maintaining high accuracy, which largely decreased the computational costs and plasma biomarker test costs. Additionally, we conducted a feature matching technique to align the features of the research target dataset with those of an external dataset, allowing our trained model to be evaluated on the external dataset without retraining. Our machine learning model exhibited highly accurate performance results on both the ADNI and CNTN datasets, so it generalizes well.

Distinguishing AD from other forms of dementia is difficult at present as diagnosis usually relies on cognitive assessments only. The new generation of AD therapies targets Aβ and its deposits, in particular. These drugs are likely to only work on brains that contain Aβ deposits. The work described here, which predicts which patient brains are Aβ positive, could therefore be of great value in determining which patients would benefit from these drugs, as well as helping identify different forms of dementia.

### 6.1 Limitations and future work

#### 6.1.1 Dataset bias concerns

This study faces limitations regarding dataset representativeness and generalizability that warrant careful consideration. The ADNI cohort, while valuable for research purposes, exhibits substantial demographic homogeneity that may limit the clinical applicability of our findings. Specifically, ADNI participants are predominantly well-educated, Caucasian individuals from high-resource healthcare settings, with systematic underrepresentation of racial and ethnic minorities, lower socioeconomic groups, and individuals with limited educational backgrounds. This demographic skew introduces potential algorithmic bias that could result in reduced model performance or increased prediction errors when applied to more diverse patient populations.

The implications of this bias extend beyond simple performance metrics. Different demographic groups may exhibit varying baseline biomarker levels, genetic polymorphisms affecting biomarker expression, and distinct disease progression patterns.

Furthermore, the clinical characteristics of ADNI participants may not reflect real-world patient presentations. ADNI enrolls individuals who are generally healthier, more cognitively intact, and more compliant with study protocols than typical patients presenting to memory clinics. This selection bias may result in an overestimation of model performance when applied to more heterogeneous clinical populations with comorbidities, medication effects, and varying levels of cognitive impairment.

#### 6.1.2 Model fragility and missing biomarker challenges

The performance degradation observed in the CNTN dataset reveals a vulnerability in our modeling approach that extends beyond the specific case of missing Aβ42/40 ratios. While we identified the Aβ42/40 ratio as the most important feature through random forest analysis, the model's dependence on this single biomarker exposes a fragility that could limit clinical utility. When this key biomarker is unavailable - whether due to laboratory constraints, cost considerations, or technical failures - the model's performance drops substantially, undermining its practical applicability.

The observed performance difference between ADNI (AUC 0.95) and CNTN (AUC 0.90) datasets, while numerically favorable, masks underlying model instability. The fact that performance can vary substantially based on feature availability suggests that our model may not be sufficiently robust for widespread clinical deployment.

#### 6.1.3 Future research directions

Addressing these limitations requires a multi-faceted approach that extends beyond simple dataset expansion. Future work should prioritize multi-cohort validation studies that specifically include diverse demographic groups, with particular attention to underrepresented populations. This should include collaboration with international research consortia to validate model performance across different healthcare systems and patient populations.

The development of robust imputation methods for missing biomarkers represents a critical research priority. Advanced techniques such as multiple imputation, matrix factorization, or deep learning-based approaches could potentially maintain model performance even when key biomarkers are unavailable. However, such approaches require careful validation to ensure they do not introduce additional bias or reduce prediction accuracy.

Longitudinal validation studies are essential to understand how model performance changes over time and across different disease stages. This includes assessment of prediction stability, biomarker trajectory modeling, and validation of the model's utility for disease monitoring in addition to diagnostic classification.

The development of standardized protocols for plasma biomarker measurement and quality control represents another critical research need. This includes harmonization of analytical platforms, establishment of reference standards, and development of quality assurance procedures that can be implemented across diverse clinical settings.

Finally, comprehensive health economic analyses are needed to establish the cost-effectiveness of our approach compared to current diagnostic standards. This should include assessment of downstream clinical outcomes, healthcare resource utilization, and patient quality of life measures to fully evaluate the clinical utility of plasma biomarker-based AD diagnosis.

## Data Availability

The original contributions presented in the study are included in the article/supplementary material, further inquiries can be directed to the corresponding author.

## The Alzheimer's disease neuroimaging initiative

Data used in preparation of this article were obtained from the Alzheimer's Disease Neuroimaging Initiative (ADNI) database (adni.loni.usc.edu). As such, the investigators within the ADNI contributed to the design and implementation of ADNI and/or provided data but did not participate in analysis or writing of this report. A complete listing of ADNI investigators can be found at: http://adni.loni.usc.edu/wp-content/uploads/how_to_apply/ADNI_Acknowledgement_List.pdf.
